# Fatty acid mobilization from adipose tissue is mediated by CD36 posttranslational modifications and intracellular trafficking

**DOI:** 10.1172/jci.insight.147057

**Published:** 2021-09-08

**Authors:** Alexes C. Daquinag, Zhanguo Gao, Cale Fussell, Linnet Immaraj, Renata Pasqualini, Wadih Arap, Askar M. Akimzhanov, Maria Febbraio, Mikhail G. Kolonin

**Affiliations:** 1 The Brown Foundation Institute of Molecular Medicine, University of Texas Health Science Center, Houston, Texas, USA.; 2 Department of Dentistry, University of Alberta, Edmonton, Alberta, Canada.; 3 Rutgers Cancer Institute of New Jersey, Rutgers New Jersey Medical School, Newark, New Jersey, USA.; 4 Division of Cancer Biology, Department of Radiation Oncology, and; 5 Division of Hematology/Oncology, Department of Medicine, Rutgers New Jersey Medical School, Newark, New Jersey, USA.; 6 Department of Biochemistry and Molecular Biology, University of Texas Health Science Center, Houston, Texas, USA.

**Keywords:** Cell Biology, Metabolism, Adipose tissue, Cancer, Obesity

## Abstract

The mechanism controlling long-chain fatty acid (LCFA) mobilization from adipose tissue is not well understood. Here, we investigated how the LCFA transporter CD36 regulates this process. By using tissue-specific KO mouse models, we showed that CD36 in adipocytes and endothelial cells mediated both LCFA deposition into and release from adipose tissue. We demonstrated the role of adipocytic and endothelial CD36 in promoting tumor growth and chemoresistance conferred by adipose tissue–derived LCFAs. We showed that dynamic cysteine S-acylation of CD36 in adipocytes, endothelial cells, and cancer cells mediated intercellular LCFA transport. We demonstrated that lipolysis induction in adipocytes triggered CD36 deacylation and deglycosylation, as well as its dissociation from interacting proteins, prohibitin-1 (PHB) and annexin 2 (ANX2). Our data indicate that lipolysis triggers caveolar endocytosis and translocation of CD36 from the cell membrane to lipid droplets. This study suggests a mechanism for both outside-in and inside-out cellular LCFA transport regulated by CD36 S-acylation and its interactions with PHB and ANX2.

## Introduction

Overgrowth and dysfunction of white adipose tissue (WAT) in obesity is associated with type 2 diabetes and other life-threatening diseases, including cancer (1–5). WAT is composed of adipocytes, the lipid-storing cells that, via endothelial cells (ECs) lining the vasculature, can either store or release lipids in response to physiological cues (5). Type 2 diabetes and other metabolic diseases, as well as some cancers, are aggravated by excessive mobilization of lipids from adipocytes. Specifically, WAT surrounding carcinomas locally drives cancer progression (6, 7). Adipocyte lipolysis is observed during advanced stages of many carcinomas (8), and recent evidence indicates a key role of adipocyte-derived fatty acids hijacked by cancer cells (9, 10). Long-chain fatty acids (LCFAs) confer cancer cells with metastatic properties and chemoresistance (11). Adipocytes adjacent to tumors clearly serve as a source of LCFAs for cancer cells (12–14). Contribution of LCFAs mobilized into the circulation from WAT not directly in contact with tumors may also contribute at advanced cancer stages when lipolysis becomes systemic.

Short- and medium-chain fatty acids (under C_12_) can passively diffuse across the cell membrane, although LCFA uptake is facilitated by a high-affinity, low-capacity receptor system (15, 16). The fatty acid transport protein (FATP) and the membrane fatty acid–binding protein (FABP) families (17), as well as caveolins (18), participate in the multiple steps of fatty acid trafficking. Cellular LCFA uptake is facilitated by fatty acid translocase (FAT), also known as CD36 (19, 20). However, the role of CD36 in lipolysis has remained unclear, and molecular mechanisms regulating LCFA mobilization from adipocytes are not well understood. Both ECs and adipocytes express CD36 (21). Studies with mice lacking CD36 systemically (19) or in ECs (22) demonstrated that CD36 is required for normal uptake of LCFAs in various organs. There is evidence that CD36 also plays a role in lipolysis (23, 24). However, mice lacking CD36 in adipocytes have not been reported. Therefore, the importance of CD36 for LCFA release from WAT in vivo and how the FAT complex switches from lipid import to export have remained unknown.

CD36-dependent lipid transport operates in the context of lipid rafts, the cell membrane microdomains with distinct sterol/sphingolipid content (25, 26). We previously reported that CD36 forms a complex with 2 other lipid raft–associated proteins, prohibitin-1 (PHB) and annexin A2 (ANX2), in adipocytes and the endothelium of WAT. We also demonstrated that these interacting proteins are localized to the plasmalemma in the presence of extracellular LCFAs (27). It has been shown that CD36 localization to lipid rafts and the cell membrane depends on glycosylation (21). In addition, CD36 subcellular localization is regulated by S-acylation, a covalent modification of the 4 cysteines in the intracellular domains of CD36 with LCFAs, such as palmitate (28, 29). Palmitoylation of PHB upon cell exposure to extracellular LCFAs has also been reported (30). ANX2 translocation to lipid rafts is also induced by LCFAs (31), but there has been no published evidence for S-acylation of ANX2.

Here, we used genetic mouse and cell culture models to investigate how CD36 regulates intercellular LCFA transport under lipolytic conditions. Our data indicated that CD36, in both ECs and adipocytes, is a gatekeeper of LCFA release from WAT, and that inhibition of its function in adipocytes or the endothelium results in reduced LCFA mobilization and bioavailability for tumors. We showed that lipolysis induction resulted in reduction of CD36 S-acylation and glycosylation, its dissociation from plasmalemma and from PHB and ANX2, and its caveolae-associated trafficking to lipid droplets.

## Results

### Both adipocytic and endothelial CD36 regulate LCFA transport in WAT.

**Figure 1:**
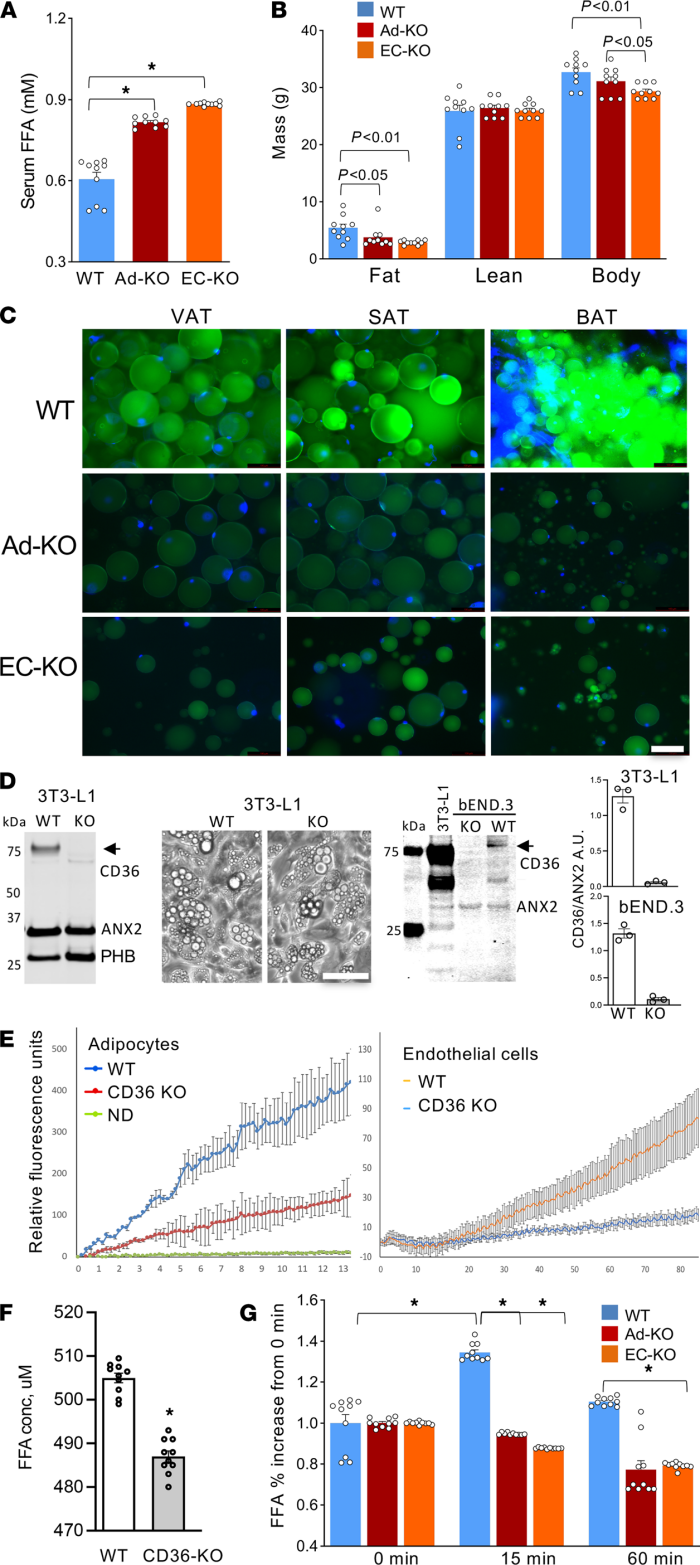
CD36 in adipocytes and the endothelium mediates LCFA transport in adipose tissue. (**A**) Steady-state plasma concentration of free fatty acids (FFAs), higher in CD36 EC-KO and Ad-KO mice. *n* = 10 mice. (**B**) Body composition measured with EchoMRI and body weight (BW) revealed reduced adiposity in CD36-KO mice (*n* = 10 mice, 1-way ANOVA). (**C**) Reduced BODIPY-C_16_ uptake by adipocytes in CD36 Ad-KO and EC-KO mice. Visceral adipose tissue (VAT), subcutaneous adipose tissue (SAT), and brown adipose tissue (BAT) were recovered 180 minutes after i.v. BODIPY-C_16_ injection and green fluorescence was imaged in cell suspension upon tissue digestion with collagenase. Scale bar: 50 μm; blue: DNA. (**D**) Western blotting confirmed KO of CD36 in 3T3-L1 cells. Images: lipid droplet formation in control and CD36-KO adipocytes differentiated for 5 days. Western blotting on proteins extracted from control and CD36-KO bEND.3 cells demonstrated loss of CD36, but not of PHB and ANX2, immunoblotted for as loading controls. Arrow: glycosylated CD36. Arrowhead: nonglycosylated CD36. Graphs: Western quantification in AU. Scale bar: 50 μm for all panels. (**E**) QBT assay demonstrating that LCFA uptake by CD36-null 3T3-L1 adipocytes and bEND.3 cells was inefficient compared with nondifferentiated (ND) cells. *n* = 5 wells. (**F**) Concentration of FFA in culture medium 3 hours after lipolysis induction in WT and CD36-null 3T3-L1 adipocytes (*n* = 10 wells, Student’s *t* test). (**G**) Relative increase in plasma concentration of FFA 15 minutes after isoproterenol injection observed in WT but not CD36-KO mice (*n* = 10 mice, 1-way ANOVA). In all panels, data are shown as mean ± SEM; *P < 0.01.****

As a model to analyze CD36 function in vivo, we used the adiponectin-Cre (Apn-Cre) mouse strain with adipocyte-specific Cre expression (32) and the Tie2e-Cre mouse strain with endothelium-specific Cre expression (33). We crossed these mice with CD36^fl/fl^ mice to generate Apn-Cre CD36^fl/fl^ (Ad-KO), TIE2e-Cre CD36^fl/fl^ (EC-KO), and Cre-negative CD36^fl/fl^ (WT) littermates. Plasma analysis in nonfasted mice revealed elevated steady-state levels of free fatty acids (FFAs) in CD36 Ad-KO and EC-KO mice, consistent with an expected alteration in lipid flux (Figure 1A). Compared with WT mice, Ad-KO and EC-KO mice displayed reduced body fat accumulation (Figure 1B). To determine whether the adipose tissue phenotype is due to abnormal LCFA uptake, we i.v. injected mice with a fluorophore-labeled palmitic acid, BODIPY-FL-C_16_, and analyzed adipocytes recovered from adipose tissue after 180 minutes of circulation. BODIPY-FL-C_16_ accumulation was markedly lower in adipocytes from visceral, subcutaneous, and brown adipose tissue in CD36 Ad-KO and EC-KO mice compared with WT controls (Figure 1C). To further investigate the consequences of CD36 deficiency, we analyzed triglyceride and cholesterol profiles in CD36 EC-KO mice. Levels of VLDL triglycerides were 1.4 times higher in males and 1.8 times higher in females; LDL and HDL triglycerides were also elevated in females compared with WT controls (Supplemental Figure 1A; supplemental material available online with this article; https://doi.org/10.1172/jci.insight.147057DS1). For total cholesterol, there was a 7% decrease in females and a 15% decrease in males, with LDL cholesterol decreased by 23% (Supplemental Figure 1B). Consistent with reduced LCFA uptake by adipocytes indicated by circulating fatty acid increase (Figure 1C), there was a compensatory effect on carbohydrate metabolism. Analysis of mice raised on chow revealed increased glucose clearance in male and female CD36 EC-KO mice (Supplemental Figure 1C), indicating increased reliance on glucose as an energy substrate. There was no significant difference in insulin sensitivity between EC-KO and WT mice (Supplemental Figure 1D). Combined, these results indicated that CD36 was required for normal CD36 transport in adipocytes and ECs of adipose tissue.

### CD36 in adipocytes and the endothelium mediates LCFA mobilization.

Next, we established cell culture models to investigate the function of CD36. We used CRISPR/Cas9 to knock out CD36 in 3T3-L1 adipocytes and bEND.3 ECs (Figure 1D). Immunoblotting confirmed CD36 loss in both cell lines (Figure 1D). Adipocytes lacking CD36 underwent normal lipogenesis upon adipocyte differentiation induction, as evident from lipid droplet formation (Figure 1D). This indicated that, at least in high-glucose medium, carbohydrates were sufficient lipogenic substrates. The QBT assay demonstrated that LCFA uptake by CD36-null 3T3-L1 adipocytes and bEND.3 EC was inefficient compared with control cells (Figure 1E). This indicates that both cell types rely on CD36 for LCFA transport. Because CD36 was previously implicated in lipolysis (24), we also used the cell culture model to analyze the role of CD36 in LCFA mobilization. Upon lipolysis induction, CD36-null 3T3-L1 adipocytes released FFAs into the medium significantly less efficiently than WT adipocytes (Figure 1F). We also used CD36 Ad-KO and EC-KO mice to analyze LCFA mobilization in vivo. Upon lipolysis induction by injection of a β-adrenergic agonist, isoproterenol, circulating FFA levels were expectedly increased in WT mice. In contrast, circulating FFA induction was not observed in CD36 Ad-KO or EC-KO mice (Figure 1G). This indicated that CD36 in adipocytes and ECs facilitated fatty acid release from WAT into the circulation.

### CD36 in cancer cells is not required for LCFA uptake from adipocytes.

**Figure 2:**
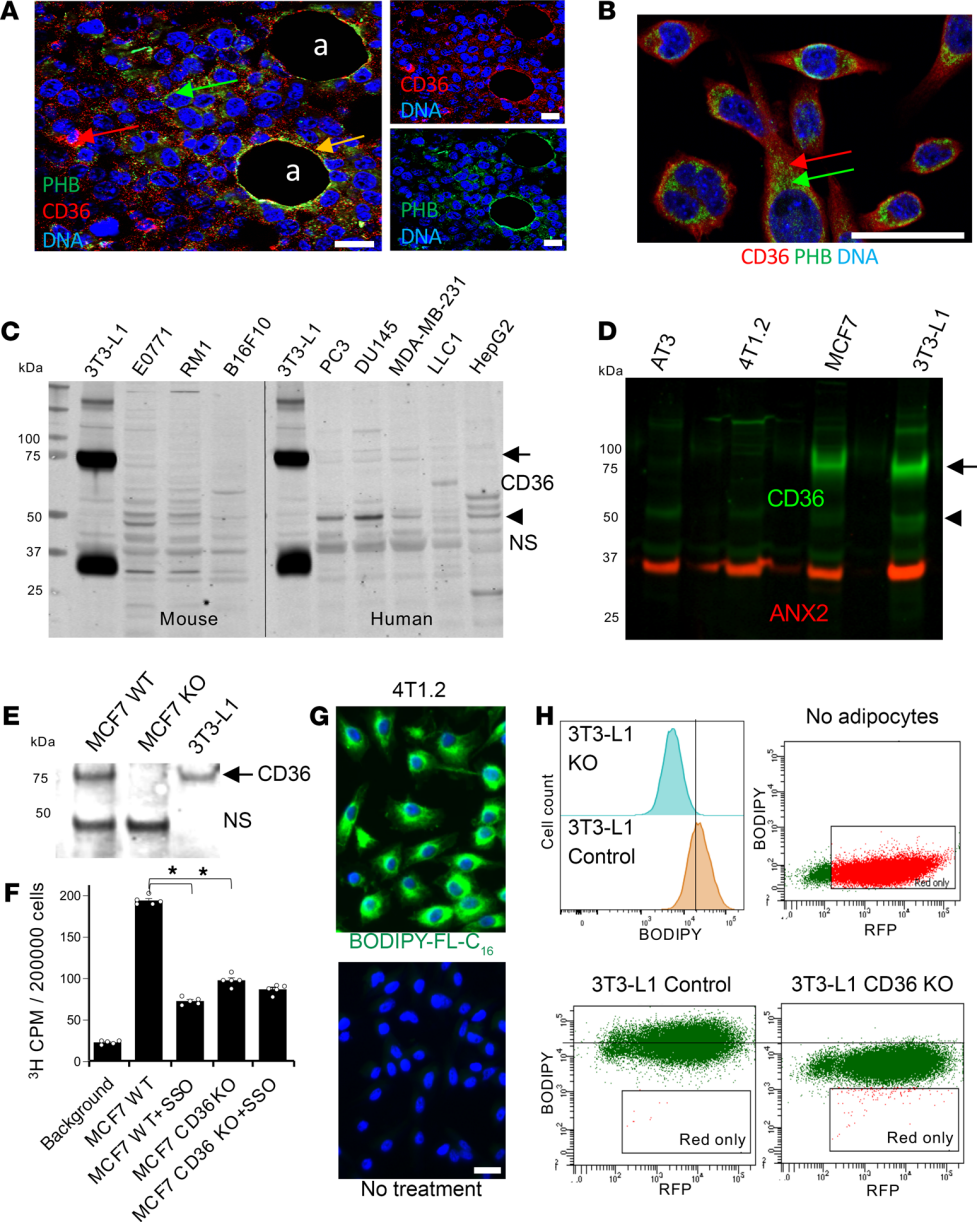
CD36 in cancer cells is not required but can promote LCFA transport. (**A**) Immunofluorescence (IF) on paraffin sections of E0771 tumor grafts showing mainly intracellular CD36 and PHB expression in cancer cells; in intratumoral adipocytes (a) colocalization is at the surface (yellow arrow). Blue: nuclei. (**B**) IF showing that CD36 and PHB (arrows) are intracellular in cultured E0771 cells. Blue: nuclei. (**C**) Western blotting on extracts from murine and human cell lines demonstrated low expression of CD36 in cancer cells. Arrow: glycosylated CD36. Arrowhead: nonglycosylated CD36. NS, nonspecific band. (**D**) Western blotting demonstrated expression of CD36 in MCF7 cancer cells comparable to that in 3T3-L1 adipocytes. Arrow: glycosylated CD36. Arrowhead: nonglycosylated CD36. ANX2 immunoblotting: loading control. (**E**) Western blotting confirming CD36 KO by CRISPR/Cas9 in MCF7 cells transduced with sgCD36. NS, nonspecific band. (**F**) ^3^H CPM in indicated cell cultures after 30 minutes exposure to 75 μM ^3^H-palmitate demonstrated that ^3^H-palmitate uptake was inhibited by SSO and CD36 KO. *n* = 5 independent wells. Data are shown as mean ± SEM; *P < 0.01, (1-way ANOVA). (**G**) 4T1.2 cells preinduced to undergo lipogenesis were untreated or treated with BODIPY-FL-C_16_ for 10 minutes and imaged for LCFA uptake (arrow). (**H**) Intercellular fatty acid transfer from 3T3-L1 adipocytes (not plotted) preloaded with BODIPY-FL-C_16_ (green) to cocultured RFP^+^ 4T1 cells detected by flow cytometry with 530 nm (BODIPY) and 610 nm (RFP) lasers. The histogram shows the difference in BODIPY-FL-C_16_ uptake for double-positive (BODIPY-FL-C_16_^+^/RFP^+^) 4T1 cells cocultured with WT versus CD36-KO adipocytes. Scale bar: 50 μm.

To model the transfer of LCFA from adipocytes to cancer cells, we analyzed carcinoma cell lines. Analysis of E0771 breast tumor grafts showed that, although CD36 and PHB colocalized at the surface of intratumoral adipocytes, they were mainly intracellular in cancer cells (Figure 2A). The lack of CD36 and PHB colocalization at the cell surface was confirmed in E0771 cell culture (Figure 2B). In protein extracts, Western blotting revealed approximately 75 kDa glycosylated and lower molecular weight bands (Figure 2, C and D). The approximately 50 kDa band, predominant in some of the cancer cell lines, is likely to be nonglycosylated CD36, as confirmed by PNGase F treatment (Supplemental Figure 2A). Analysis of cancer cells demonstrated that expression of glycosylated CD36 was very low in the majority of mouse and human cancer cell lines (Figure 2C). This accounted for low CD36 cell surface presentation and posed a problem in using these cell lines to study CD36-mediated LCFA transport. An exception was MCF7, a human breast cancer cell line found to express glycosylated CD36 (Figure 2D). To assess the role of CD36 in LCFA uptake in cancer cells, we knocked out the *CD36* gene in MCF7 cells by using the CRISPR/Cas9 method and confirmed the loss of expression by Western blotting (Figure 2E). The uptake of radiolabeled 9,10-3^H^-palmitic acid (^3^H-palmitate) was significantly lower in CD36-KO MCF7 cells compared with cells transduced with a control sgRNA (Figure 2F). Treatment with sulfo-*N*-succinimidyl oleate (SSO), a competitive LCFA-CD36 binding blocker (24, 34), similarly reduced palmitate uptake (Figure 2F), confirming that LCFA uptake in cancer cells was promoted by CD36 in MCF7 cells.

To assess whether CD36 is required for LCFA uptake, we used a murine breast carcinoma cell line, 4T1.2, in which glycosylated CD36 was not detectable (Figure 2D). The uptake of BODIPY-FL-C_16_ by 4T1.2 cells that had been induced to pre-form lipid droplets in adipogenic medium was efficient, indicating that it occurred irrespective of CD36 (Figure 2G). Based on the observation that CD36 mediated fatty acid mobilization from adipocytes (Figure 1, F and G), we investigated the importance of adipocytic CD36 in LCFA transport to cancer cells. We preloaded CD36-WT and -KO adipocytes with BODIPY-FL-C_16_ and then cocultured them with 4T1.2 cells expressing a red fluorescent protein (RFP). Flow cytometry enabled quantification of BODIPY-FL-C_16_ (with a 530 nm range laser) that was scavenged from adipocytes in cancer cells gated based on expressing RFP (identified with a 610 nm range laser). BODIPY-FL-C_16_^+^/FRP^+^ cells were detected only upon incubation with BODIPY-FL-C_16_–loaded adipocytes (Figure 2H). There was a strong BODIPY fluorescence detected for 4T1.2 cells incubated with adipocytes preloaded with BODIPY-FL-C_16_ (Figure 2H). Importantly, the majority of 4T1.2 cells cocultured with CD36-WT adipocytes had BODIPY-FL-C_16_ fluorescence above the highest level detected for 4T1.2 cells cocultured with CD36-null adipocytes (Figure 2H). Together, these data indicated that CD36 function in adipocytes was more important for intercellular LCFA transport than its function in cancer cells.

### CD36 in adipocytes and the endothelium controls bioavailability of LCFA for tumors.

**Figure 3:**
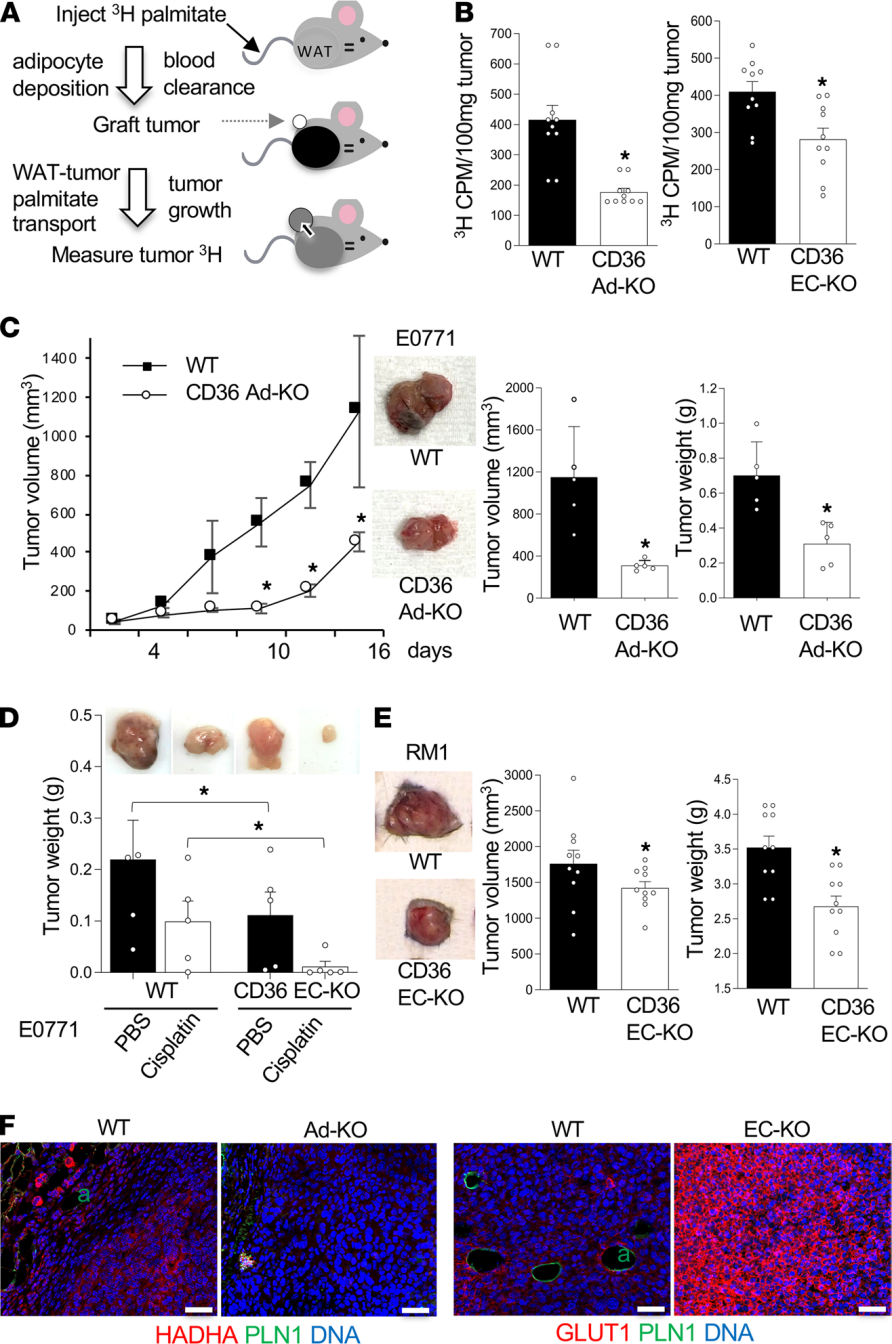
CD36 in adipocytes and ECs mediates LCFA transfer to tumors. (**A**) Experiment schematic: i.v.-administered ^3^H-palmitate was deposited into adipocytes and cleared from circulation, after which tumor cells were grafted and ^3^H content in tumors was measured by liquid scintillation counting. (**B**) ^3^H counts per minute (CPM) in 100 mg of E0771 tumor tissue recovered from mice treated as described in **A**, indicating reduced ^3^H-palmitate transfer to cancer cells in CD36 Ad-KO (left graph) and EC-KO (right graph) mice compared with CD36^+^ (WT) littermates. *n* = 10 tissue probes (Student’s *t* test). (**C**) Reduced E0771 graft growth in CD36 Ad-KO females compared with CD36^+^ (WT) littermates. *n* = 10 (Student’s *t* test). Images of representative tumors are shown in the right. (**D**) E0771 graft growth, reduced by cisplatin treatment, was further reduced in CD36 Ad-KO females compared with CD36^+^ (WT) littermates. *n* = 5 (1-way ANOVA). Images: representative tumors. (**E**) Images of RM1 tumors 3 weeks after s.c. grafting into male mice. Graphs: quantification of final tumor volume, being lower in CD36 EC-KO compared with CD36^+^ (WT) littermates. *n* = 8–10 (Student’s *t* test). (**F**) IF analysis showing that tumors in CD36 EC-KO mice lacked intratumoral adipocytes (a) positive for perilipin-1 (PLN1), lacked HADHA expression, and had increased GLUT1 expression in cancer cells. Data are shown as mean ± SEM; *P < 0.05. Scale bar: 50 μm.

To investigate the role CD36 plays in LCFA transport from WAT to tumors in vivo, we used CD36 Ad-KO and EC-KO mice and i.v. administered ^3^H-palmitate. Biodistribution of ^3^H-palmitate in tissues can be measured by liquid scintillation counting (35, 36). First, we demonstrated that upon i.v. administration, ^3^H-palmitate was deposited in adipose tissue depots and that after 1 hour it accumulated in WT and CD36-KO mice to similar levels (Supplemental Figure 3A). After 2 days, circulating ^3^H-palmitate levels were undetectable, whereas adipose tissue still contained the stored labeled palmitate (Supplemental Figure 3B). We confirmed that systemic mobilization of ^3^H-palmitate can be induced by measuring it in the blood 1 day after i.v. isoproterenol administration (Supplemental Figure 3C). To analyze the transfer of palmitate from WAT to tumors, we designed an approach described in Figure 3A. Mice were injected with 6 μCi of ^3^H-palmitate to enable its maximal accumulation in WAT. After 2 days, upon clearance of the circulating probe, tumor cells were s.c. grafted. Two weeks later, grown (~1 cm^3^) tumors were recovered and analyzed for ^3^H content by liquid scintillation counting. This confirmed that labeled palmitate was transferred to tumors (Figure 3B). By performing this assay in parallel in WT and CD36 Ad-KO littermates, we revealed that palmitate transfer to tumors was significantly lower in the absence of adipocyte or endothelial CD36 (Figure 3B). These results indicated that CD36 in adipocytes and ECs promoted LCFA release from WAT and that the systemically mobilized LCFAs were utilized by cancer cells.

The functional contribution of WAT-derived LCFAs was reflected in tumor growth. E0771 tumors orthotopically grafted into female mice grew significantly slower in CD36 Ad-KO mice compared with WT littermates (Figure 3C). A significant reduction in E0771 tumor growth was also observed for CD36 EC-KO mice compared with WT littermates (Figure 3D). A similar result was obtained in male mice grafted with RM1 prostate carcinoma. Both tumor size and tumor weight were lower in CD36 EC-KO mice compared with WT littermates (Figure 3E). Endothelial CD36 deletion also resulted in reduced growth of mammary E0771 tumors in female mice (Figure 3D and Supplemental Figure 3D). Obesity induced by a high-fat diet (HFD) prior to tumor grafting resulted in increased tumor growth for WT and CD36 EC-KO littermates (Supplemental Figure 3E). However, obesity did not make tumor growth dependency on CD36 more prominent compared with chow-fed mice (Figure 3D). This finding suggests that HFD feeding promoted tumor growth in part through CD36-independent mechanisms.

We also analyzed tumors to assess the repercussions of LCFA shortage for cancer cells. Intratumoral adipocytes, present in tumors of WT mice, were not observed for CD36 EC-KO or Ad-KO mice (Supplemental Figure 3F). This is consistent with fewer fatty acids being available for the tumors to drive intratumor adipogenesis reported previously (7). Compared to WT mice, cancer cells of KO mice had no detectable expression of hydroxyacyl-CoA dehydrogenase (HADHA), the enzyme catalyzing the last 3 steps of mitochondrial fatty acid β-oxidation (Figure 3F). In contrast, expression of GLUT1, the main tumor glucose transporter, was markedly increased in both CD36-KO models (Figure 3F and Supplemental Figure 3F). These results indicated that the absence of CD36-mediated LCFA mobilization, causing a shortage of this energy substrate, resulted in cancer cells switching from fatty acid β-oxidation to glucose metabolism. Furthermore, we performed cell culture studies to pinpoint the repercussions on cell metabolism. By using a 3T3-L1 adipocyte/cancer cell coculture model, we measured the oxygen consumption rate (OCR) upon mitochondrial transport inactivation. A Seahorse real-time ATP rate assay was performed on E0771 cells in a Transwell chamber sharing medium with adipocytes. Coculture with CD36-KO adipocytes resulted in cancer cells switching toward glycolysis at the expense of mitochondrial oxidation for ATP production (Supplemental Figure 3G). This effect on respiration is consistent with fatty acids derived from CD36-expressing adipocytes contributing to β-oxidation utilized by cancer cells.

CD36 expression and a switch to fatty acid metabolism in tumors have been linked with increased resistance to chemotherapy (9, 11). We therefore tested whether the blockade of LCFA transport might potentiate chemotherapy efficacy by treating female mice grafted with E0771 tumors with cisplatin. Cisplatin reduced tumor growth by only 2-fold in WT mice, whereas in CD36 EC-KO mice treated with cisplatin, tumor growth was almost completely blocked (Figure 3D and Supplemental Figure 3D). Combined, these results suggested that LCFA mobilization from WAT, assisted by adipocytic and endothelial CD36, contributed to cancer aggressiveness.

### CD36 posttranslational modification mediates LCFA transport in adipocytes.

**Figure 4:**
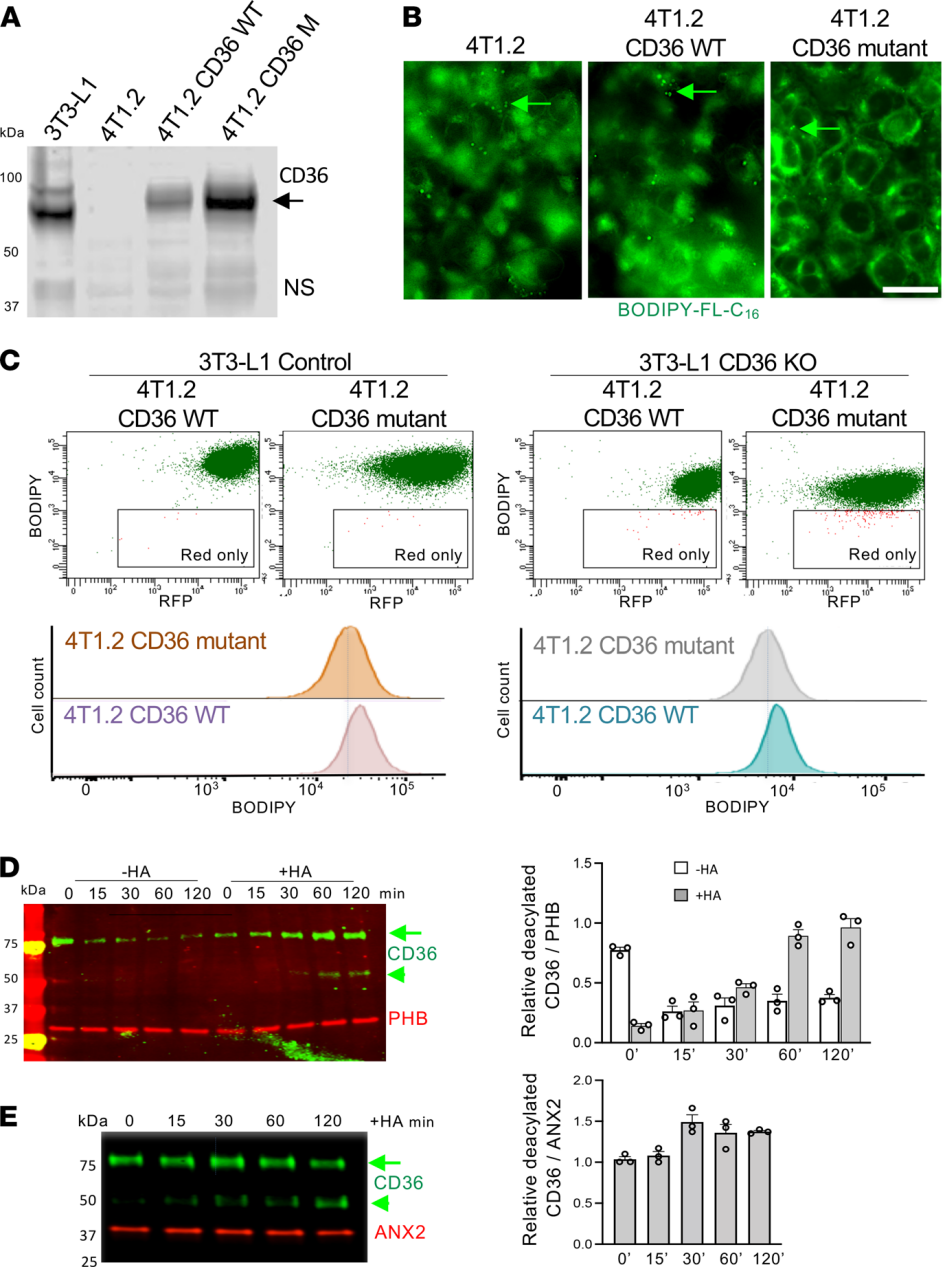
Acylation/deacylation of CD36 mediates LCFA transport. (**A**) Western blotting on extracts from 4T1.2 cells nontransduced or transduced with WT CD36 or CD36 mutant lacking S-acylated cysteines. Arrow: glycosylated CD36, also expressed in 3T3-L1 adipocytes. NS, nonspecific band. (**B**) 4T1.2 cells transduced with WT CD36 or CD36 mutant lacking S-acylated cysteines grown in 2D were preinduced to undergo lipogenesis as in Figure 2G and then treated with BODIPY-FL-C_16_ for 10 minutes and imaged to visualize uptake by lipid droplets (arrows). Scale bar: 50 μm. (**C**) Intercellular fatty acid transfer from 3T3-L1 adipocytes (not plotted) preloaded with BODIPY-FL-C_16_ (green) to adjacent RFP^+^ 4T1 cells detected by flow cytometry with 530 nm (BODIPY) and 610 nm (RFP) lasers. Histograms at the bottom are provided to compare double-positive (BODIPY-FL-C_16_^+^/RFP^+^) population in 4T1 cells expressing WT versus mutant CD36. (**D**) 3T3-L1 adipocytes treated with 300 μM palmitic acid for 0 to 120 minutes analyzed by acyl biotin exchange (ABE) assay (Supplemental Figure 4A). Extracted proteins were alkylated with hydroxylamine (HA) where indicated, de–S-acylated, biotinylated, and removed with streptavidin beads. Remaining proteins were immunoblotted with indicated antibodies. Note progressive accumulation of nonacylated glycosylated (arrow) and nonglycosylated (arrowhead) CD36 upon LCFA treatment. PHB immunoblot indicates constant PHB acylation and equal loading. Quantification is on the right. (**E**) bEND.3 endothelial cells treated with 300 μM palmitic acid for 0 to 120 minutes analyzed by ABE assay. Extracted proteins were alkylated (HA), de–S-acylated, biotinylated, and removed with streptavidin beads. The remaining proteins were immunoblotted with indicated antibodies. Note accumulation of nonacylated glycosylated (arrow) and nonglycosylated (arrowhead) CD36 upon LCFA treatment. ANX2 immunoblot indicates constant ANX2 acylation and equal loading. Quantification is on the right.

It has been demonstrated that plasma membrane localization and the LCFA transport function of CD36 rely on cysteine S-acylation with LCFAs, such as palmitate (37). In adipocytes, palmitoyl acyltransferases DHHC4 and DHHC5 have been shown to acylate CD36 (38). In adipocytes this state is dynamic, and CD36 deacylation by thioesterase APT1 mediates endocytic CD36-mediated LCFA trafficking to lipid droplets (39). To investigate the role of CD36 S-acylation in LCFA uptake by cancer cells, we used a murine breast carcinoma cell line. We transduced it with an expression plasmid to generate 4T1.2 cells expressing CD36. In parallel, a CD36 mutant in which the 4 N- and C-terminal cysteines are changed to alanines, which disables protein incorporation into lipid rafts (29), was also expressed in 4T1.2 cells. Immunoblotting confirmed that WT and mutant CD36 were glycosylated and therefore localized to the plasma membrane in these cells (Figure 4A). WT CD36 expression in 4T1.2 cells conferred them with a rounded morphology, whereas cysteine-mutant CD36 expression resulted in tighter adherence than that observed for parental cells, as evident from phalloidin staining of cytoskeleton (Supplemental Figure 2B). Upon treatment of cells with palmitic acid, CD36-dependent differences in adhesion were still observed (Supplemental Figure 2B). We also used 4T1.2 cells induced to preform lipid droplets in adipogenic medium. Lipogenesis occurred irrespective of WT or mutant CD36 expression and the response to palmitate added to the medium was not affected by CD36 expression (Supplemental Figure 2C). Upon incubation of these cells with BODIPY-FL-C_16_, we compared LCFA uptake with that observed for parental 4T1.2 cells. We also did not detect an obvious effect of WT or mutant CD36 expression on the amount and intracellular distribution of BODIPY-FL-C_16_ (Figure 4B).

To establish the importance of CD36 acylation in intercellular LCFA transport, we preloaded CD36-WT and -KO adipocytes with BODIPY-FL-C_16_, cocultured them with 4T1.2 cells expressing WT or cysteine-mutant CD36, and analyzed them by flow cytometry. Consistent with data in Figure 2H, transfer to both 4T1.2 variants from CD36-KO adipocytes was lower than from CD36-WT adipocytes (Figure 4C). However, BODIPY-FL-C_16_ uptake by cancer cells was only marginally higher upon WT CD36 expression compared with cysteine-mutant CD36-expressing cancer cells (Figure 4C). This result reinforces our conclusions from Figure 2, indicating that in cancer cells CD36 was not rate limiting for LCFA uptake and that CD36 function was more important for cancer cell cytoskeletal organization and adhesion.

To investigate the role of CD36 acylation in cells of WAT, we used the acyl-biotin exchange (ABE) assay (Supplemental Figure 4A) based on a previously established approach (40). Consistent with previous reports (28–30), we detected S-acylation of CD36 and PHB in adipocytes (Figure 4D). Interestingly, these experiments also revealed S-acylation of ANX2 (Figure 4E), which has not been previously reported, to our knowledge. These 3 interacting proteins were found to be acylated in 3T3-L1 adipocytes (Figure 4D) and ECs (Figure 4E). Consistent with a recent report on LCFAs inducing CD36 deacylation and internalization (39), CD36 S-acylation was decreased in both cell types in response to palmitate treatment (Figure 4, D and E). In contrast to CD36, there was no detectable change in S-acylation of ANX2 or PHB in response to palmitic acid treatment (Figure 4, D and E). Glycosylation of CD36 is also known to be required for its cell membrane localization (21). Concomitantly with deacylation, the fraction of nonglycosylated 50 kDa CD36 was also increased by palmitate treatment (Figure 4, D and E). These results suggest that posttranslational modifications of CD36 mediate its functions in various cells engaged in LCFA transport.

### CD36 deacylation mediates fatty acid release from adipocytes.

**Figure 5:**
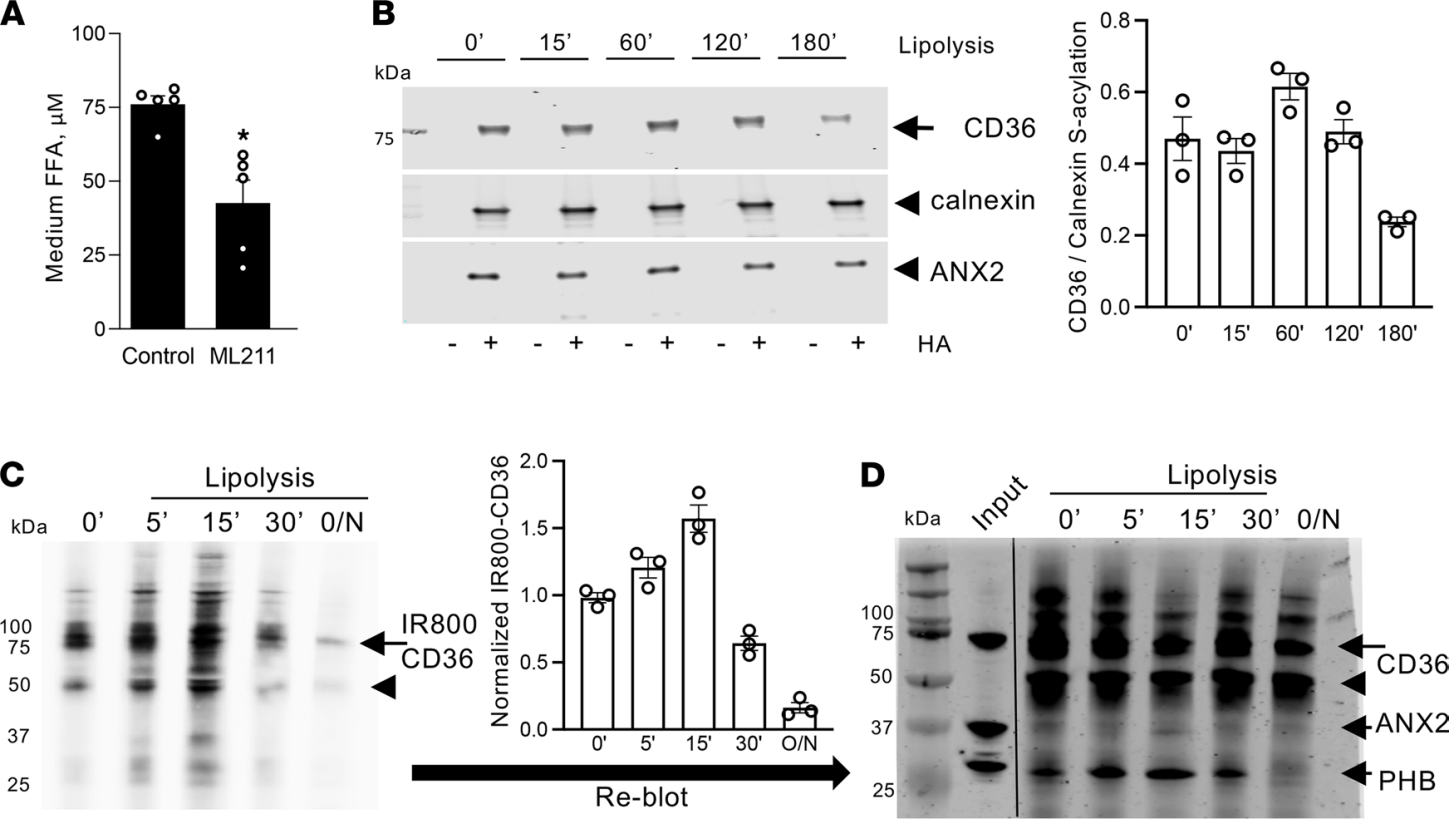
CD36 deacylation induced by lipolysis. (**A**) Concentration of FFA in culture medium 5 hours after lipolysis induction in 3T3-L1 adipocytes untreated or treated with an inhibitor of S-acylation, ML211 (30 nM). Data are shown as mean ± SEM; *P < 0.05 (Student’s *t* test). (**B**) ABE assay (Supplemental Figure 4A) on 3T3-L1 1adipocytes untreated or induced to undergo lipolysis for indicated time (minutes). Note that IP of S-acylated CD36, specifically observed upon hydroxylamine (HA) treatment, was partly inhibited by lipolysis. Immunoblotting for calnexin and ANX2 from the same extracts indicated constant ANX2 acylation and equal loading. Quantification is on the right. (**C**) CD36 metabolic labeling (Supplemental Figure 4B). After incubation of live 3T3-L1 adipocytes with alkynylated LCFA analog (0.1 mM 17-ODA, 12 hours), de novo S-acylation of CD36 was detected by IP with anti-CD36 antibodies, subsequent click chemistry with IRDye800-azide probe (IRDye800-N3), and SDS-PAGE. Note that IP of S-acylated CD36, specifically observed upon HA treatment, was inhibited by lipolysis induction (IBMX/forskolin/isoproterenol). Arrow: glycosylated CD36. Arrowhead: nonglycosylated CD36. CD36-IR800 quantification is on the right. (**D**) Probing of the IP blot from **C** with PHB and ANX2 antibodies, and subsequently with CD36 antibodies, demonstrated a decrease in PHB and ANX2 association with CD36 concomitant with a decrease of CD36 acylation upon lipolysis induction.

To investigate the importance of S-acylation during LCFA mobilization from adipose tissue, we used a thioesterase inhibitor, ML211, which inhibits both APT1 and APT2 (41). By measuring FFA concentration in the culture medium 3 hours after lipolysis induction in 3T3-L1 adipocytes, we demonstrated that concomitant treatment with ML211 significantly inhibited fatty acid release from adipocytes (Figure 5A), suggesting that deacylation mediated LCFA mobilization. We then used the ABE assay to analyze S-acylation of specific proteins upon lipolysis induction in adipocytes. A gradual decrease in CD36 S-acylation was observed 2 hours after treatment with isoproterenol/IBMX/forskolin (Figure 5B). In contrast, there was no detectable change in S-acylation on ANX2 or calnexin (Figure 5B). To complement ABE, we used the metabolic labeling assay (Supplemental Figure 4B) based on LCFA analogs that can be labeled with a fluorophore to measure de novo S-acylation of proteins (42, 43). That assay revealed that de novo S-acylation of CD36 was also decreased by both LCFA treatment and lipolysis induction (Supplemental Figure 4C). A time course demonstrated that lipolysis induction resulted in a gradual decrease of CD36 S-palmitoylation after 30 minutes (Figure 5C). To investigate the response of the PHB/ANX2/CD36 complex to lipolytic stimuli, we used co-IP. Immunoblotting with PHB and ANX2 antibodies demonstrated that the gradual decrease in de novo CD36 S-palmitoylation was concomitant with a decrease of CD36 presence in the complex containing PHB and ANX2 observed after 30 minutes of lipolysis induction (Figure 5D), as quantified in Supplemental Figure 4D. These data suggest that CD36 deacylation underlies its function in lipolytic conditions.

### Lipolysis induces CD36 trafficking to lipid droplets.

**Figure 6:**
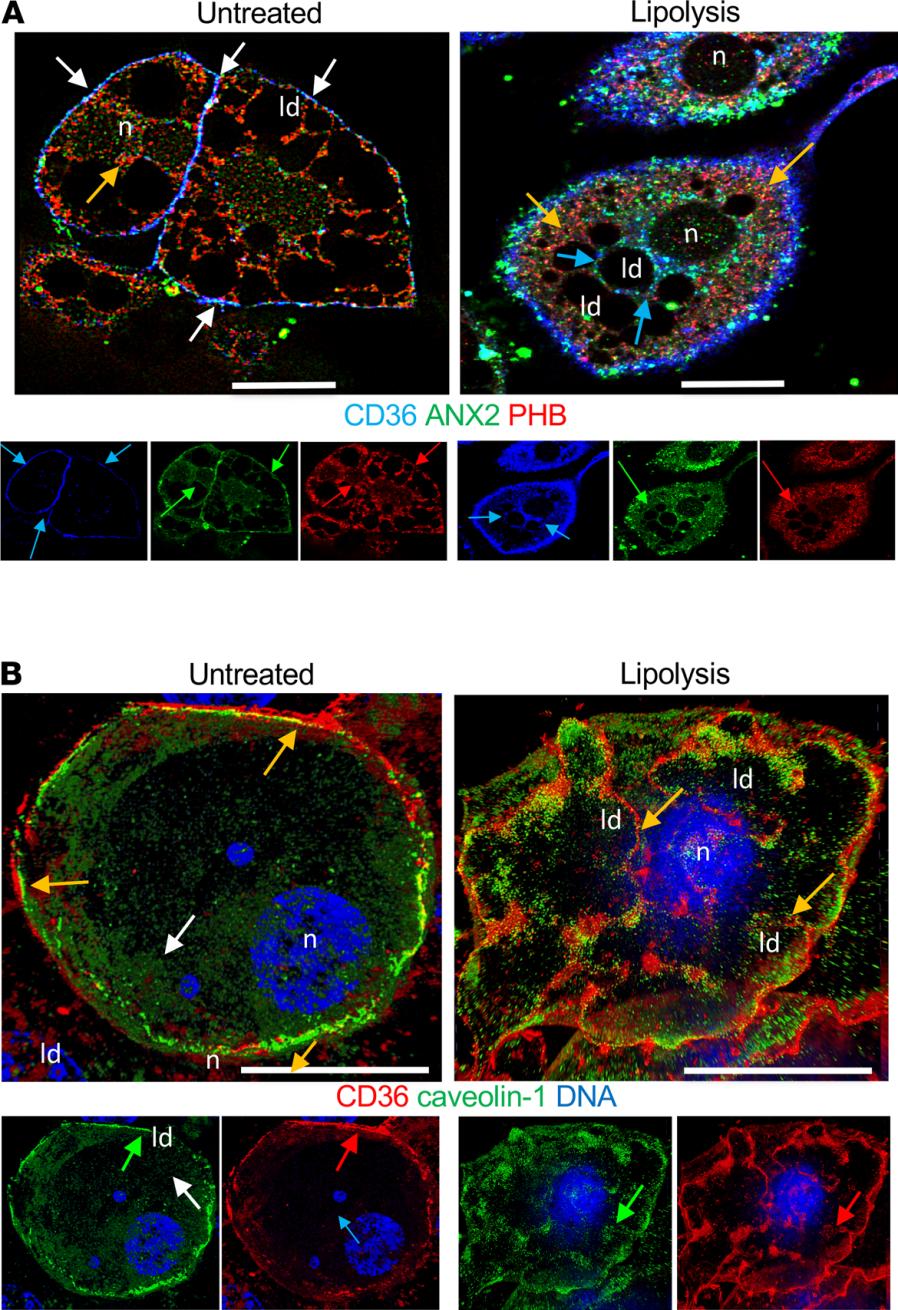
Lipolysis induces CD36 trafficking to lipid droplets. (**A**) Immunofluorescence analysis of 3T3L1 adipocytes with antibodies against indicated proteins and secondary antibodies conjugated with green, red, and blue fluorophores. Note that in control adipocytes, CD36 was concentrated on the cell membrane along with PHB and ANX2 (white arrows), whereas 30-minute treatment with lipolysis-inducing agents induced CD36 dissociation from PHB (red arrows) and ANX2 (green arrows), internalization (blue arrows), and localization to lipid droplets (ld). n, nuclei. (**B**) Immunofluorescence analysis of 3T3L1 adipocytes with antibodies against indicated proteins and secondary antibodies conjugated with green and red; nuclei (n) are stained blue. Note that in control adipocytes, CD36 was concentrated on the cell membrane along with caveolin-1, whereas 30-minute treatment with lipolysis-inducing agents induced translocation of CD36 and caveolin-1 to ld.

**Figure 7:**
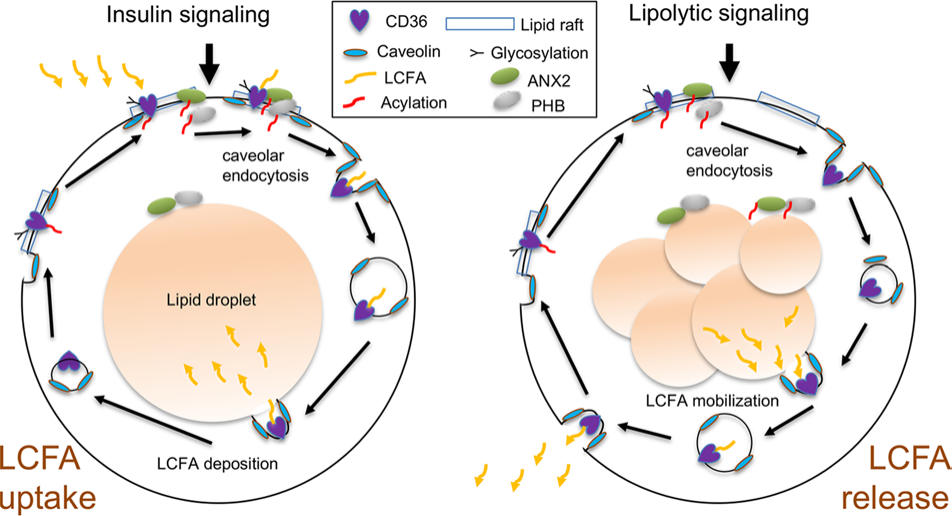
A model of CD36-mediated outside-in and inside-out LCFA transport. Postprandially, insulin signaling activates extracellular LCFA uptake mediated by acylated CD36 at the cell surface in complex with PHB and ANX2. Deacylation of CD36 enables caveolar endocytosis and lipid droplet trafficking of LCFA-bound CD36. In lipolytic conditions, CD36 deacylation, caveolar endocytosis, and lipid droplet trafficking enable CD36 loading with LCFA released from lipid droplets and their plasma membrane trafficking and release via exocytosis.

Finally, we used confocal immunofluorescence to analyze cellular localization of the FAT complex proteins in 3T3-L1 adipocytes. In unstimulated adipocytes, CD36 was observed mainly at the cell membrane, where it colocalized with ANX2 and PHB (Figure 6A) as well as with caveolin-1 (Figure 6B). Upon lipolysis induction, plasmalemma dissociation and translocation of CD36 toward lipid droplets was observed within 30 minutes (Figure 6A). Although ANX2 and PHB were concomitantly dissociated from the plasma membrane, their colocalization with CD36 was reduced intracellularly (Figure 6A). In contrast, lipid droplet–translocated CD36 remained colocalized with caveolin-1 upon lipolysis induction (Figure 6B). Based on our observations and the reported role of caveolar CD36 endocytosis in LCFA trafficking to lipid droplets (39), we propose a model according to which LCFA mobilization is similarly regulated by CD36 deacylation and trafficking to lipid droplets followed by exocytotic release of LCFAs from cells (Figure 7).

## Discussion

A better understanding of the mechanisms regulating lipid storage in WAT and lipid trafficking is important for improving approaches to treat metabolic diseases and cancer. Although CD36 is known as a key receptor facilitating LCFA uptake (20, 44), molecular events mediating systemic LCFA release from WAT and biodistribution in lipolytic conditions have remained poorly understood (16). Here, we used mouse and cell culture models to investigate the importance and function of CD36 in adipocytes, the endothelium, and cancer cells for LCFA mobilization from adipocytes and LCFAs’ intercellular transfer.

CD36 is a multifunctional protein expressed in various cell types (21). The global KO mouse model has revealed a CD36 requirement for normal triacylglycerol deposition in WAT (19). A report on EC-specific CD36 KO confirmed the importance of endothelial CD36 for LCFA transport from the circulation (22). However, it has remained unclear how LCFAs are mobilized from WAT and traffic to other tissues. Here, we characterized an adipocyte-specific CD36 KO and performed a side-by-side phenotypic analysis of CD36 EC-KO and Ad-KO mice. We showed that CD36 in both cell types was required for efficient LCFA deposition into adipocytes. The difference in the effect of EC and Ad CD36-KO on adiposity could be due to the endothelium relying on CD36 function systemically. We also showed that CD36 in adipocytes and ECs promotes LCFA release into the circulation upon lipolysis induction. A metabolic phenotype similar to that of Adn-Cre CD36^fl/fl^ mice was observed for another adipocyte-specific CD36-KO model, the aP2-Cre CD36^fl/fl^ mouse model, corroborating our results (data not shown). Previous reports suggested a role of CD36 in lipolysis (21, 23, 24, 45). Reinforcing that notion, here we demonstrated that CD36 in adipocytes and ECs was rate limiting not only for LCFA uptake but also for LCFA release from WAT.

CD36 is known to serve as a receptor for lipoproteins and LCFAs (19, 35, 46–49). The mechanism through which CD36 mediates transport in and out of the cell has remained unclear (20, 34, 44). CD36 has been proposed to act by increasing concentration of lipids at the cell surface and facilitating their diffusion across the phospholipid bilayer (20, 34, 44, 50). However, this model has remained debated (51). We showed that a reduction in CD36 S-acylation was a hallmark of both LCFA uptake and LCFA release upon lipolysis induction. Previously, glycosylation and S-acylation of CD36 has been shown to enable its cell surface localization (21, 29, 39). A recent study has shed light on the role of APT enzymes, as well as of palmitoyl acyltransferases DHHC4 and DHHC5 in controlling CD36 acylation, its caveolar endocytosis, and trafficking LCFAs to lipid droplets (39). Extending this observation, we showed that CD36 S-acylation was reduced after treatment with LCFA not only in adipocytes but also in ECs. Importantly, our results indicated that lipolysis induction also induced CD36 deacylation by thioesterases as well as its deglycosylation, dissociation from the cell membrane, and trafficking to lipid droplets.

Our data indicated that dynamics in S-acylation and glycosylation were linked with CD36 subcellular trafficking and changes in its interactions with ANX2 and PHB. We had previously discovered that CD36 function to uptake LCFAs is supported by its cell surface interaction with ANX2 and PHB proteins (27). Here, we identified S-acylation of ANX2 and found that, like PHB, it was constantly S-acylated in adipocytes. Interestingly, our data revealed that CD36 dissociated from ANX2 and PHB inside the cell upon lipolysis induction and its translocation to lipid droplets. Our data suggest a mechanistic model according to which posttranslational modification and subcellular localization of CD36 enable its LCFA transport function in 2 alternative processes, LCFA uptake and LCFA release from adipocytes (Figure 7). We propose that both LCFA uptake upon insulin signaling and LCFA mobilization upon lipolytic signaling are mediated by the dynamic state of CD36 S-acylation. Deacylation of CD36 enabled its trafficking to either deposit LCFAs into lipid droplets or to take LCFA to the cell surface for release.

Finally, our data indicated that CD36-mediated LCFA mobilization from WAT stimulated tumor growth and enabled resistance to chemotherapy. Activation of lipid metabolism in cancer is linked with metastases and therapy resistance (11, 52, 53). In cancer cells, fatty acids are utilized for sterol, sphingolipid, phospholipid, and triglyceride synthesis, lipid modification of proteins, and fatty acid β-oxidation to serve as a source of energy (54, 55). Fatty acids can be generated by endogenous de novo lipogenesis from other energy sources (54), but uptake of exogenous fatty acids plays an important role in cancer (56). Increased usage of external fatty acids by cancer cells is observed in aggressive carcinomas (14). WAT is the main lipid reservoir of the body, and there is building evidence that LCFAs from lipolytic adipocytes become hijacked by cancer cells (9). Revealing this, carcinoma growth is associated with lipolysis in adipocytes (7, 13, 57, 58). Conversely, inhibition of lipolysis lowers circulating FFA levels and reduces cancer pathogenicity (55). Recent studies suggest that the CD36 on the cancer cell surface plays a role in LCFA uptake (11, 59). Elevated CD36 expression is linked with enhanced aggressiveness in carcinomas and sarcomas (13, 60, 61). CD36 expression has been linked with increased resistance to chemotherapy (9, 11) and with increased lipid metabolism, metastasis, and poor prognosis (11, 62). Although CD36 expression in cancer cells is low, its expression, induced by adipocyte contact, has been shown to drive cancer progression (63). Our data revealed that the majority of mouse and human cancer cell lines lack CD36 glycosylation, a modification enabling cell membrane localization (51), aside from expressing relatively little CD36. These results indicate that in cancer cells, LCFA uptake occurs largely through a CD36-dependent mechanism, and that, consistent with a previous report (64), CD36 function in cancer cells is more important for cytoskeletal organization and cell adhesion. As our data demonstrated, it is CD36 in adipocytes and the endothelium that is a gatekeeper of LCFA transfer from WAT to tumors. We showed that CD36-mediated release of LCFA from WAT predetermined the reliance of cancer cells for β-oxidation as opposed to glycolysis and their increased chemoresistance. Because CD36 also plays a role in angiogenesis and inflammation (21), alterations in these processes in the endothelial KO and their potential effect on cancer progression cannot be excluded. In addition, changes in the transport of cholesterol, also regulated by CD36, could affect tumor growth. The multifaceted roles of CD36 in linking lipid metabolism and cancer progression are yet to be further investigated.

In summary, our study provides an important insight on how dynamic posttranslational modifications of CD36, its interaction with ANX2 and PHB, and subcellular endocytic and exocytic translocation mediate LCFA trafficking intracellularly. We also demonstrated that CD36 activity in the endothelium and adipocytes regulated systemic LCFA biodistribution. Continuation of this work of dissecting the details of the underlying molecular mechanisms and testing the role of CD36 in mobilizing other types of lipids will outline new directions for intervention in metabolic disease and cancer.

## Methods

### Animal experiments.

We used previously described Tie2e-Cre (33, 65) and CD36^fl/fl^ (66) mouse strains and Apn-Cre (32) stock 010803 purchased from The Jackson Laboratory. All mice were in C57BL/6 background and were crossed and genotyped as described (66). Mice were housed in the animal facility with a 12-hour light/12-hour dark cycle at room temperature and had free access to water and diet. The HFD used was 58 kcal% fat (Research Diets, D12331). Physiological tests were performed as we previously described (10, 27, 67). Body composition (fat and lean mass) was measured by using EchoMRI-100 (Echo Medical Systems). Blood glucose concentration was measured with a glucometer (One Touch Ultra). Lipolysis was induced by i.p. injection of isoproterenol (10 mg/kg) into overnight-fasted mice. Plasma FFAs were measured with a kit from MilliporeSigma (50-489-265). BODIPY-C_16_ (Molecular Probes) was i.v. injected (0.1 mg). Radiolabeled LCFA in indicated tissues was measured upon i.v. injection of 6 μCi of 9,10-3H(N)-palmitic acid (NET043001MC, PerkinElmer) by liquid scintillation counting by using Wallac 1209 RackBeta (LKB) as described (35, 36). For tumor studies, 1 × 10^5^ cancer cells were grafted with a 21-gauge needle into the mammary fat pad (E0771) or subcutaneously onto the upper flank (RM1) as described (68). Cisplatin (VWR 95031-032) was i.p. injected at the dose of 10 mg/kg once a week. Tumor size was measured with a caliper weekly, and volume was calculated as length × width^2^ × 0.52. Tumors were weighed upon resection. For adipose tissue analyses, after heart perfusion with 10 mL PBS, inguinal adipose tissue, gonadal adipose tissue, and interscapular brown adipose tissue were resected, minced, and digested in 0.5 mg/mL collagenase type I (Worthington Biochemical) solution under gentle agitation for 1 hour at 37°C, and cover-slipped suspensions were imaged within 30 minutes as described (10, 27, 69, 70).

### Cell lines and culture assays.

Cells were cultured in DMEM/10% FBS. 3T3-L1 (CL-173), bEND.3 (CRL-2299), B16F10 (CRL-6475), PC3 (CRL-1435), DU145 (HTB-81), MDA-MB-231 (HTB-26), LLC1 (CRL-1642), HepG2 (HB-8065), and MCF7 (HTB-22) were purchased from American Type Tissue Collection. 4T1.2 cells expressing RFP were described previously (71). The other cell lines were received from their original developers: E0771 from F.M. Sirotnak (Memorial Sloan-Kettering Cancer Center, New York, New York, USA), RM1 from T.C. Thompson (University of Texas MD Anderson Cancer Center, Houston, Texas, USA), and AT3 from S.I. Abrams (Roswell Park Comprehensive Cancer Center, Buffalo, New York, USA). 3T3-L1 CD36-KO cell lines were created by CRISPR/Cas9-mediated KO. To construct *pLenti-CRISPR/Cas9 mCD36* gRNA expression vectors, target sequence 5′-TCAATAAGCATGTCTCCGACTGG-3′ containing the PAM sequence (underlined) was ligated into *Lenti-CRISPR v2* plasmid (Addgene plasmid 52961). The target sequence 5′-CGGAACTGTGGGCTCATCGCTGG was used to knock out CD36 in MCF7 cells. For expression of WT CD36 and Cyst-mutant CD36, full-length mouse CD36 cDNA was cloned into pLenti-c-MyC-DDK-puro vector (Origene, PS910092) and CD36 Cyst-mutant was generated by changing cysteines 3, 7, 464, and 466 to serines by using a quick-change site-directed mutagenesis kit (Agilent, 200522). White adipogenesis induction was performed as described (69) by growing cells to confluence in medium containing 1.7 μM insulin, 0.5 mM IBMX, and 1 μM dexamethasone/0.2 μM indomethacin for 3 days and 1.7 μM insulin afterward. BSA-emulsified palmitic acid (MilliporeSigma) was used at 50 μM. Lipolysis was induced in medium containing 50 mM IBMX, 10 μM forskolin, and 10 μM isoproterenol. SSO was used at 200 μM. For fatty acid release inhibition assay, 3T3-L1 adipocyte medium was exchanged into DMEM (low glucose) without FBS for 2 hours, after which lipolysis was initiated in the presence or absence of 30 nM ML211 (Cayman Chemical, 17630). After 3 hours, fatty acid released into the medium was measured with a fatty acid assay kit (Bioassay Systems, EFFA-100). Oil-red-O staining was performed as described (69). For coculture assays, 3T3-L1 adipocytes predifferentiated for 8 days were treated with 2 μM BODIPY-C_16_ for 30 minutes, detached with trypsin, washed, and mixed with RFP-expressing 4T1.2 cells (1:5). For flow cytometry, cells were disaggregated with trypsin; pregated to exclude debris, cell clumps, and dead cells based on DAPI staining; and quantified based on green and red fluorescence with FACSAria/FlowJo software (BD Biosciences) as described (7). LCFA uptake by adipocytes was quantified with the QBT assay (Molecular Devices); LCFA uptake was measured with a QBT assay as described (10, 27, 69, 70).

### Acylation and IP studies.

3T3-L1 adipocytes were incubated in DMEM–high glucose without FBS overnight before the assays. A metabolic labeling assay was performed as described (72). ABE assay was performed as described (43). Precipitated and flow-through samples were loaded onto a 4%–20% gradient SDS-PAGE gel (Bio-Rad) and analyzed by blotting onto Immobilon-FL membrane (MilliporeSigma), blocking with Odyssey buffer (LI-COR), and probing with specified antibodies in PBS/0.05% Triton X-100. The following antibodies were used for IP: anti-ANX2 (Abcam, Ab41803), anti-CD36 (Novus, NB400-144), and anti-PHB (MilliporeSigma, HPA003280) at 5 μg IgG/mg protein extract. The following antibodies were used for immunoblotting: anti-ANX2 (Santa Cruz Biotechnology, sc-30757, 1:1000), anti-CD36 (Novus, NB400-144, 1:3000), and anti-PHB (Santa Cruz Biotechnology, sc-18196, 1:500). Signal was detected by Odyssey CLx imaging system (LI-COR). ImageJ (NIH) was used to quantify bands.

### Immunofluorescence.

Paraformaldehyde-fixed cells were analyzed by immunofluorescence as described (7, 69). Upon blocking and permeabilization with 0.05% Tween 20, the following primary antibodies (4°C, 12 hours) and secondary antibodies (room temperature, 1 hour) diluted in PBS were used: anti-PHB (MilliporeSigma, HPA00380, 1:75); anti-ANX2 (GeneTex, GTX22242, 1:75); anti-CD36 (Bio-Rad, MCA2748, 1:75); and anti-Cave1 (R&D Systems, MAB5736, 1:100). Donkey anti-rabbit Alexa Fluor 488 (catalog A21206) and donkey anti-goat Alexa Fluor 488 (catalog A11055) were from Invitrogen, Thermo Fisher Scientific; Cy3-conjugated IgG (catalog 711-166-152, 1:300) was from Jackson ImmunoResearch. Donkey Dylight-650 secondary antibody was from Invitrogen, Thermo Fisher Scientific (catalog SA5-10169). Images of cells fixed on Nunc Lab-Tek II chamber slides or tissue preparations were acquired with super resolution IF, which was performed with a Nikon N-SIM/N-STORM microscope.

### Statistics.

GraphPad Prism software and Microsoft Excel were used to graph data as mean ± SD or SEM as indicated and to calculate *P* values by using 1-way ANOVA or homoscedastic 2-tailed Student’s *t* tests. *P* values less than 0.05 were considered statistically significant.

### Study approval.

Animal studies were approved by and performed according to the guidelines of the IACUC of the University of Texas (Houston).

## Author contributions

MGK, MF, and AA conceived and designed the experiments, analyzed data, and wrote the manuscript; RP and WA analyzed data and edited the manuscript; ZG, ACD, CF, and LI designed and performed the experiments, analyzed data, and edited the manuscript.

## Supplementary Material

Supplemental data

## Version 1 07/27/2021

In-Press Preview

## Version 2 09/08/2021

Electronic publication
