# Supplementary material for: Fatty acid mobilization from adipose tissue is mediated by CD36 posttranslational modifications and intracellular trafficking
Source: JCI Insight. 2021 Sep 8;6(17):e147057. doi: 10.1172/jci.insight.147057 (PMC8492349; doi:10.1172/jci.insight.147057)
Supplement: Supplemental data [file jciinsight-6-147057-s171.pdf]

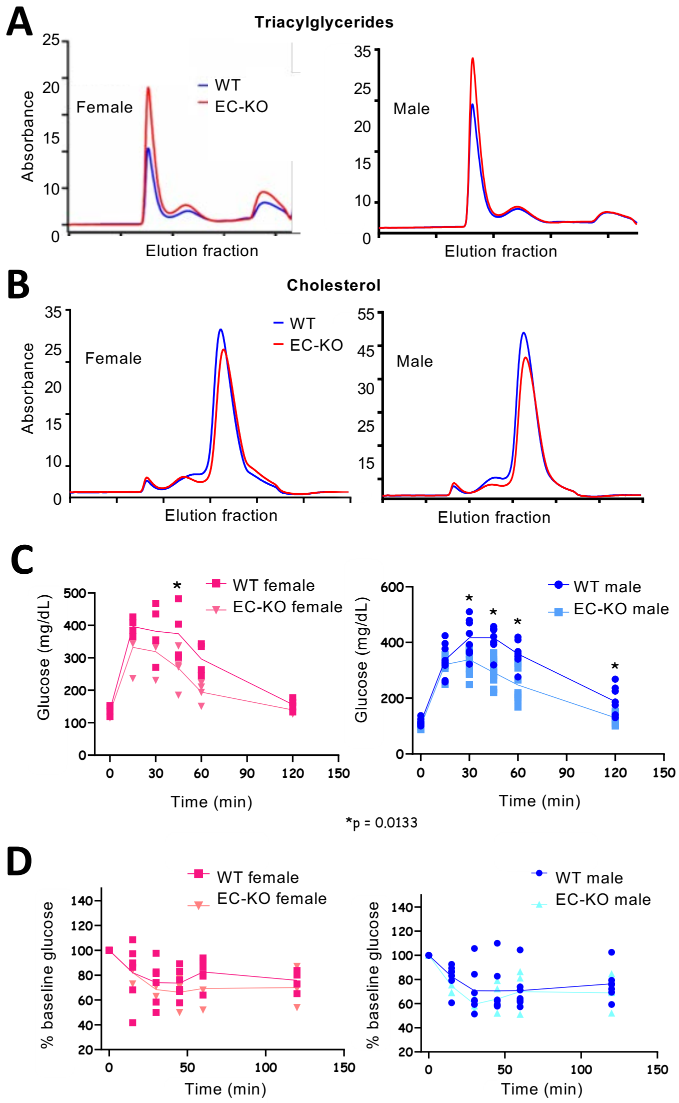

Supplemental Figure 1

Metabolism in CD36 EC-KO mice. (**A**) Plasma triglycerides: higher VLDL in both males and female EC-KO (flox/flox CD36 Tie2e-Cre+) mice, compared to WT controls (flox/flox CD36). LDL and HDL is higher in EC-KO females compared to WT controls. (**B**) Plasma cholesterol: lower in EC-KO males and females compared to WT controls. (**C**) Glucose tolerance test: overnight-fasted EC-KO and WT male and female littermates (5 month-old) raised on chow were injected with glucose (1 g/kg body weight) i.p. and glucose in blood was measured. (**D**) Insulin tolerance test: 4 hr-fasted EC-KO and WT male and female littermates (5 month-old) raised on chow were injected with insulin (0.6 U/kg body weight) i.p. and glucose in blood was measured.

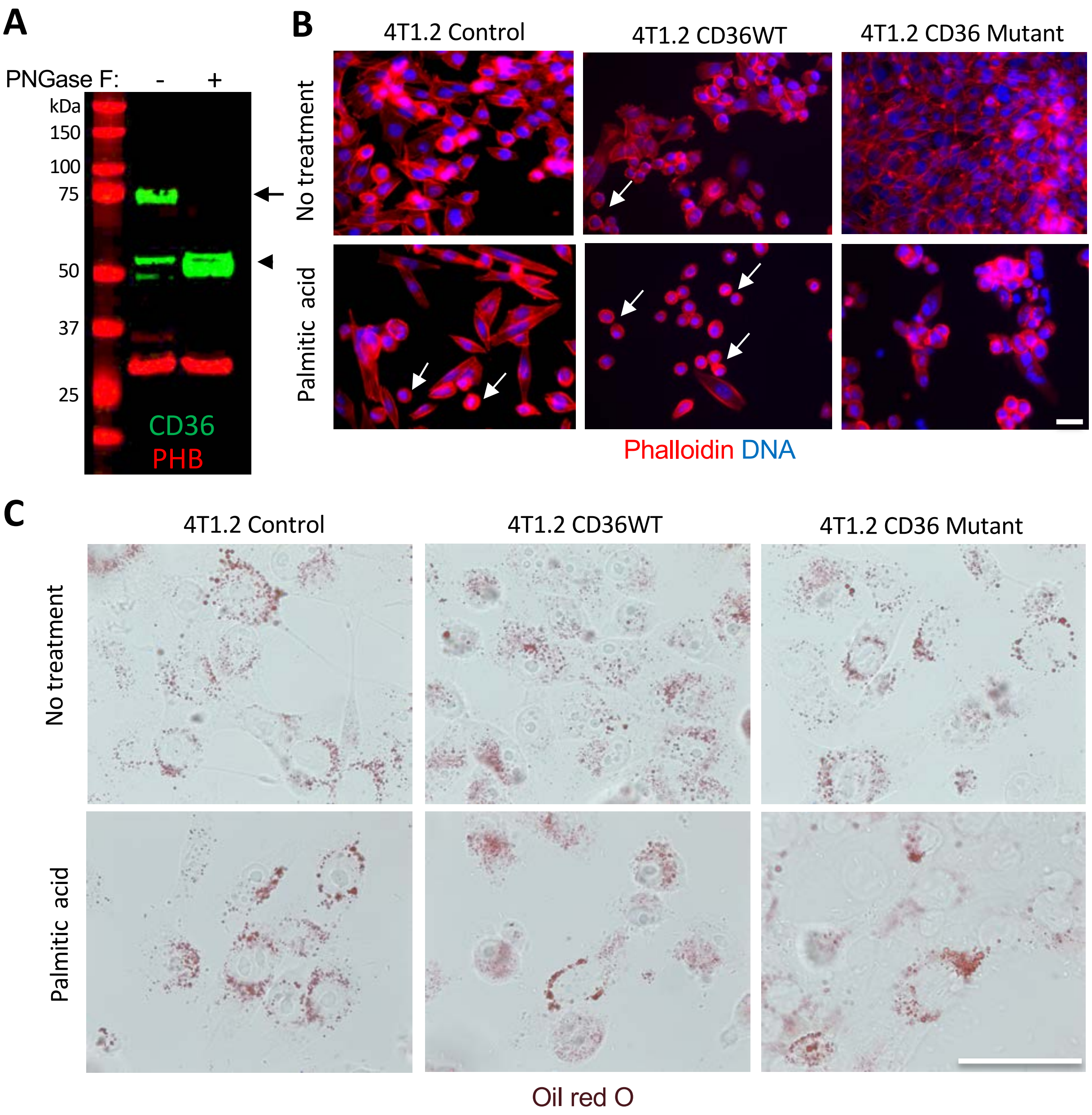

Supplemental Figure 2

The role of CD36 in LCFA uptake by cancer cells. (**A**) 3T3L1 adipocyte protein extract with and without treatment with PNGase F that removes N-linked oligosaccharides. PHB Immunoblotting is done for loading control. Arrow: glycosylated CD36. Arrowhead: non-glycosylated CD36. (**B**) Cy3-conjugaed phalloidin localization in fixed 4T1.2 cells reveals rounded cytoskeleton architecture (arrow) upon WT CD36 expression and adherent fibroblastic morphology in cells expressing CD36 mutant lacking S-acylated cysteines. 12 hr exposure to 300 µM palmitic acid promotes cell rounding due to lipid droplet formation. (**C**) 4T1.2 cells pre-induced to undergo lipogenesis were incubated with 50 µM palmitic acid overnight, fixed and stained with Oil-red-O (brown). No difference in lipid droplet formation and palmitate deposition is detected between cells expressing WT CD36, CD36 mutant lacking S-acylated cysteines, or unmodified 4T1.2 cells. Scale bar: 50 µm.

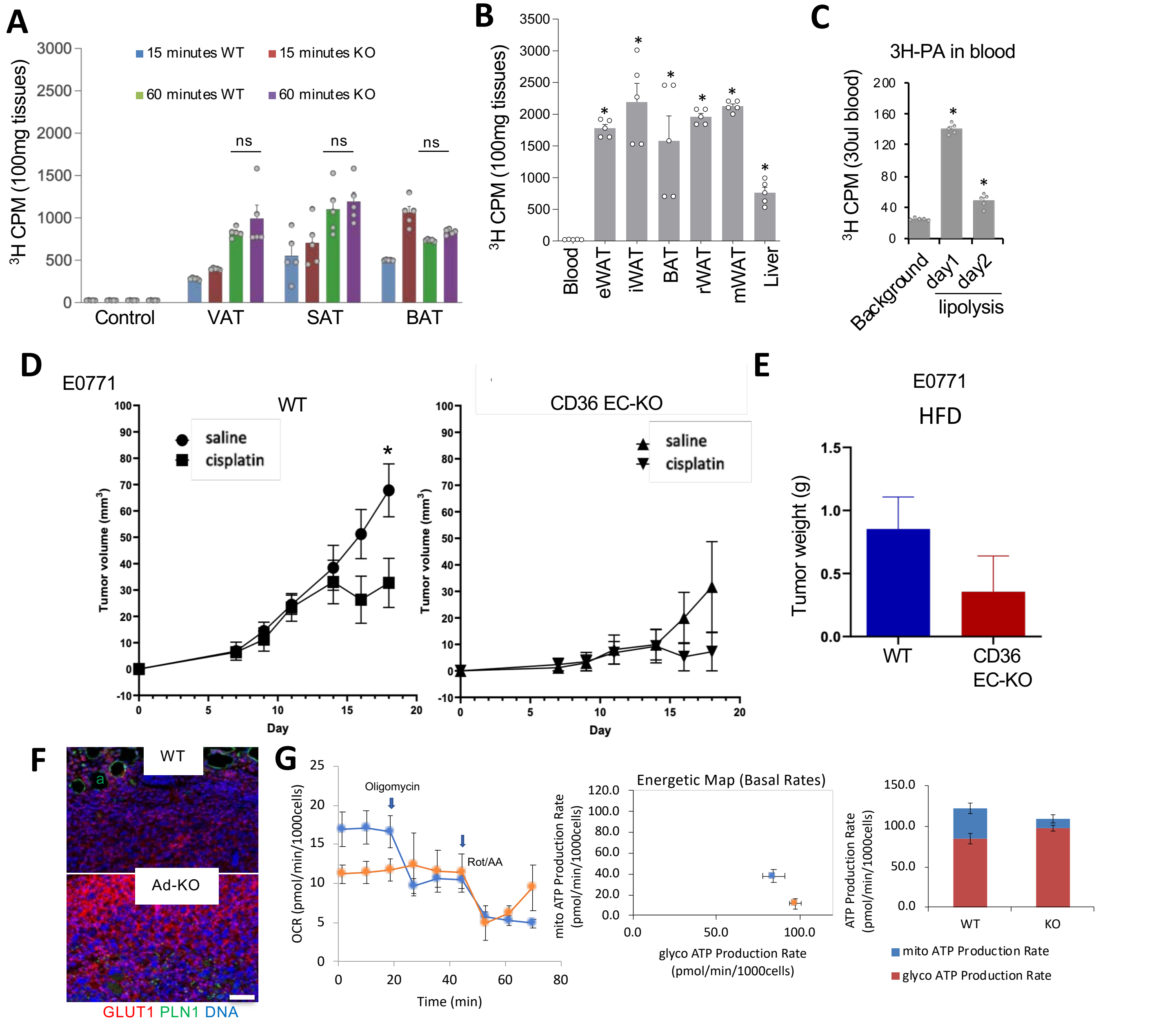

Supplemental Figure 3

CD36 in adipocytes and endothelium mediates LCFA transfer to tumors. (**A**) ^{3}H counts per minute (CPM) in 100 mg of indicated tissues recovered from WT or CD36 Ad-KO, mice 15 and 60 min after IV ^{3}H-palmitate injection in experiment described in Figure 2A. ns: not significant. Control: SAT from a mouse not injected with ^{3}H-palmitate. (**B**) ^{3}H CPM in 100 mg of indicated tissues recovered from CD36 Ad-KO mice 2 weeks after IV ^{3}H-palmitate injection in experiment described in Figure 2A. eWAT: Epididymal WAT; iWAT: inguinal WAT; BAT: interscapular BAT; rWAT: retroperitoneal WAT; mWAT: mammary WAT. (**C**) ^{3}H CPM in 30 µL of blood 2 weeks after IV ^{3}H-palmitate injection in experiment described in Figure 2A before (background) and 1-2 days after lipolysis indication by IV isoproterenol administration in CD36 Ad-KO mice. Background: blood from a mouse not injected with ^{3}H-palmitate. In **A**-**C**, N=5 independent tissue preps. (**D**) Growth curves for E0771 tumors analyzed in Figure 2D showing growth, reduced by cisplatin treatment, further reduced in CD36 Ad-EC females, compared to CD36+ (WT) littermates. N=5. (**E**) Weights of E0771 tumors grown (for 3 weeks) in WT and CD36-Ad-EC littermates obese due to HFD pre-feeding. N=5 mice. Plotted are mean ± SEM; **P*<0.05 (Student's t-test). (**F**) IF analysis showing that tumors in CD36 Ad-KO mice lack intratumoral adipocytes (a) positive for perilipin-1 (PLN1) and have increased GLU1 expression in cancer cells. Scale bar: 50 µm. (**G**) Seahorse Realtime ATP Rate assay performed on EO771 cells in a transwell chamber sharing medium with 3T3-L1 WT and CD36 KO adipocytes in 10% FBS containing 1000mg glucose/L DMEM for 48 hours. Oxygen consumption rate (OCR) was measured upon successive treatment with oligomycin (1mM), FCCP (Carbonyl cyanide-p-trifluoromethoxyphenylhydrazone, 1mM) and rotenone / antimycin A (0.5mM). Relative contributions of mitochondrial oxidation and glycolysis to ATP production are plotted on the right.

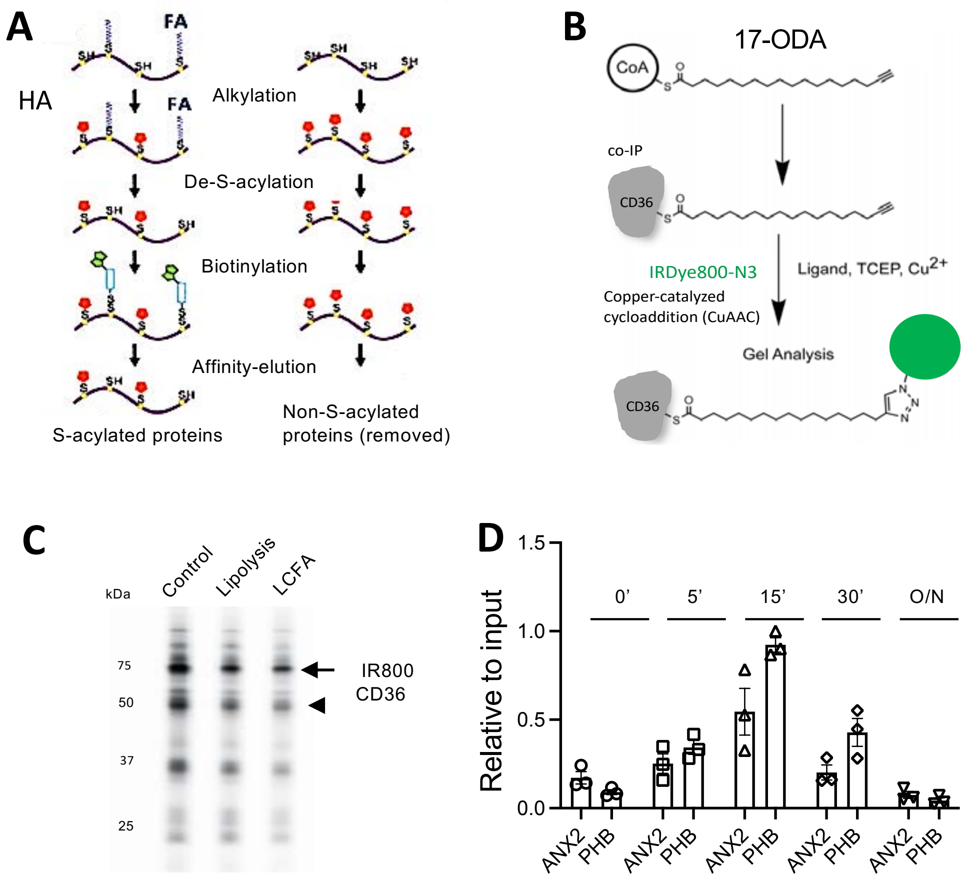

Supplemental Figure 4 Assays used to quantify FAT protein S-acylation and de-acylation
(**A**) Schematic of acyl-biotin exchange (ABE) assay, in which free cysteine thiols (-SH) are irreversibly blocked by N-ethylmalemide (NEM) following cell or tissue lysis and thioester bonds between cysteines and acyl groups are then specifically cleaved by neutral hydroxylamine (HA). The newly formed free thiol groups, once biotinylated, can be captured by streptavidin and analyzed by SDS-PAGE. (**B**) In bioorthogonal metabolic labeling assay, an alkynylated fatty acid analog is added to live cells and incorporated into native S-acylation sites. After cell lysis, CD36 is immunoprecipitated and conjugated to an azide IRDye800 fluorophore, by copper (I)-catalyzed azide-alkyne Huisgen's cycloaddition reaction ("click chemistry"). (**C**) CD36 metabolic labeling (Supplemental Figure 4B) performed for 3hr after either lipolysis induction (IBMX/forskolin/isoproterenol) or LCFA (1% Intralipid) treatment for 30 min. After incubation of live 3T3L1 adipocytes with alkynylated LCFA analog (0.1 mM 17-ODA), *de novo* S-acylation of CD36 was detected by IP with anti-CD36 antibodies, subsequent click chemistry with IRDye800-azide probe (IRDye800-N3), and SDS-PAGE. Arrow: glycosylated CD36. Arrowhead: non-glycosylated CD36. (**D**) Quantification of band intensities in Figure 5D.
